# Ferritin: An Inflammatory Player Keeping Iron at the Core of Pathogen-Host Interactions

**DOI:** 10.3390/microorganisms8040589

**Published:** 2020-04-18

**Authors:** Ana C. Moreira, Gonçalo Mesquita, Maria Salomé Gomes

**Affiliations:** 1 i3S—Instituto de Investigação e Inovação em Saúde, Universidade do Porto, 4200-135 Porto, Portugal; goncalo.mesquita@i3s.up.pt (G.M.); sgomes@ibmc.up.pt (M.S.G.); 2 IBMC—Instituto de Biologia Molecular e Celular, Universidade do Porto, 4200-135 Porto, Portugal; 3 ICBAS—Instituto de Ciências Biomédicas Abel Salazar, Universidade do Porto, 4050-313 Porto, Portugal

**Keywords:** ferritin, inflammation, infection, host-directed therapy

## Abstract

Iron is an essential element for virtually all cell types due to its role in energy metabolism, nucleic acid synthesis and cell proliferation. Nevertheless, if free, iron induces cellular and organ damage through the formation of free radicals. Thus, iron levels must be firmly controlled. During infection, both host and microbe need to access iron and avoid its toxicity. Alterations in serum and cellular iron have been reported as important markers of pathology. In this regard, ferritin, first discovered as an iron storage protein, has emerged as a biomarker not only in iron-related disorders but also in inflammatory diseases, or diseases in which inflammation has a central role such as cancer, neurodegeneration or infection. The basic research on ferritin identification and functions, as well as its role in diseases with an inflammatory component and its potential as a target in host-directed therapies, are the main considerations of this review.

## 1. Introduction

**Figure 1:**
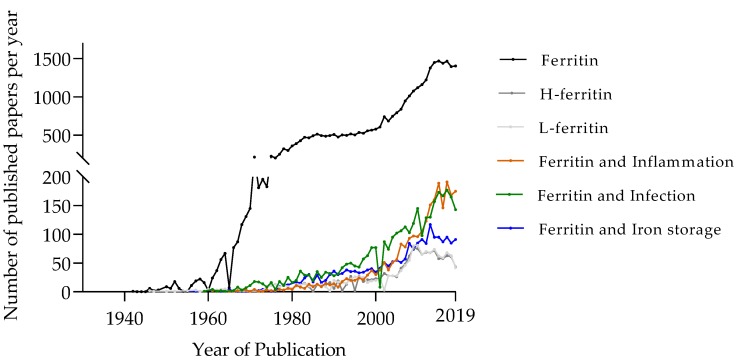
The increasing number of published papers in the PUBMED (http://www.ncbi.nlm.nih.gov/sites/entrez/) archive between 1940 and 2019. All the trends show a tendency to increase over the years. The black line above represents the number of publications found using the keyword “ferritin”. The light grey and dark grey lines below represent the number of papers found with the keywords “L-ferritin” or “H-ferritin”, respectively. Increasing numbers of articles have been found in recent years using the keywords “ferritin” and “infection” (green line) or “ferritin” and “inflammation” (brown line), while the combination of “ferritin” and “iron storage” (blue line) gives a relatively stable number of papers in the last decade.

Almost all life forms on Earth depend on iron. Due to its redox properties, this element is the most suitable co-factor for crucial proteins responsible for energy production, mitochondrial respiration, DNA and RNA synthesis and cell proliferation. However, free iron induces cell and tissue damage through the formation of free radicals. Thus, it needs to be strictly controlled and properly stored to avoid pathology. Accordingly, iron traffic and distribution in animals are controlled by complex systems, operating both at the systemic and at the cellular levels [1]. In a situation of infection, both host and pathogen need to maintain the access to the iron needed for cell survival and proliferation while avoiding its toxicity. Additionally, the animal host puts into action several mechanisms aimed at limiting the access to iron by microbes [2]. While many iron-related proteins have been acknowledged to play a role in host–pathogen interaction, in this review we will focus on ferritin. Since its discovery, the interest on ferritin has been steadily increasing in the scientific community (Figure 1). Interestingly, the increase in the number of studies describing ferritin in association with inflammation or infection has been more marked in the last 10 years than that of articles related to ferritin in iron storage. Here, we will revisit the fundamental studies that allowed ferritin identification and the elucidation of its main functions, but also discuss the role of this protein in host–pathogen interaction and its potential as a target for host-directed therapies.

## 2. Ferritin, the Iron Storage Protein: Identification and Characterization

Ferritin was one of the first proteins known to be involved in iron metabolism. It was first described by the end of the 19th century as an iron-rich component of horse liver and later purified from horse spleen in the 1930s [3,4]. Ferritin is a large, spherical molecule that stores iron inside its hollow center. It has been largely conserved throughout evolution, demonstrating the essentiality of the protein and of its functions. More than just an iron deposit, ferritin is now known to protect cells from the nefarious effects of free iron, besides being involved in different functions, including immune regulation [5,6]. Generally, ferritin is a cytosolic protein, but it has been found in mitochondria, in the nucleus and in serum [7,8]. The cytosolic ferritin consists of a polypeptide with 24 protein subunits that compose the apoferritin shell. This structure is an approximately 450kDa hollow-cage that is capable of sheltering up to 4500 Fe^3+^ atoms and is composed of two types of subunits: H-ferritin (FTH) and L-ferritin (FTL) with nearly 20kDa each [9]. In humans, the genes coding for the two proteins that make the apoferritin shell are located in different chromosomes, the H in the eleventh chromosome and the L in the nineteenth [10]. Both subunits have specific functions and have different expression levels in different tissues. The different H/L ratios can be explained by the differential regulation of their expression at the transcriptional and translational levels [11]. In 1991, the ferroxidase moiety on the FTH protein was disclosed, leading to the understanding of the specific function of this subunit of ferritin [12]. This ferroxidase activity is responsible for the conversion of Fe^2+^ to Fe^3+^, allowing ferritin to sequester iron inside the shell as a hydrous ferric oxide with a structure similar to the “ferrihydrite” [13]. This function is exclusively performed by the FTH, since the FTL does not have a ferroxidase center. FTH is essential, as mice lacking the gene coding for FTH (*Fth1*) cannot survive embryonic development [14]. FTH is the predominant subunit expressed in the heart or brain tissues, possibly due to the higher requirement of these tissues for the ferroxidase activity [15]. Although FTL lacks the iron-oxidizing capacity, it has other functions. The presence of L chains in ferritin improves the stability of the protein and helps iron incorporation [16]. Its expression is higher in spleen and in the liver [15]. The H subunits of ferritin, as stated before, are the ones that will oxidize the iron making it possible to be stored inside the shell. The Fe^2+^ enters ferritin with the help of an electrostatic gradient that attracts metal cations and, once internalized, the ferrous iron migrates to the ferroxidase center located on the heavy chain of ferritin. On this center, the Fe^2+^ atoms will react with hydrogen peroxide and will be oxidized to Fe^3+^ [17]. Ferritin is important to ensure that iron is safely stored and detoxified, preventing the oxidative damage that could be caused by reactions like Haber–Weiss or Fenton. We have recently showed that, although the conditional deletion of FTH in macrophages did not impact in vitro differentiation and physiology in basal conditions, it resulted in higher susceptibility to oxidative stress and cell death induced by exogenously added iron [18]. Besides H- and L-ferritin, which are predominantly cytosolic, early in this century, a novel ferritin was identified: mitochondrial ferritin. This peptide is synthesized as a precursor of about 30kDa, while its mature form has a molecular weight of 21-22kDa and 79% homology to FTH [7]. Its expression is limited to tissues with a high energy demand, such as brain, testis and heart and is not modulated by iron levels [3].

As previously mentioned, ferritin can also be found in the serum [19]. The functions or effects of serum ferritin are mostly unknown, but variations in serum ferritin levels can be clinically relevant. Serum ferritin is traditionally used as an indicator of tissue iron levels in the body, either to identify iron overload conditions or to distinguish between iron-deficient or non-iron-deficient types of anemia. However, it is also known to represent one of the serum markers of inflammation, and in that regard it can help the diagnosis of a variety of conditions, including autoimmune diseases, Still’s disease, neurologic disorders or cancer [20]. In the next section, we will review the state of the art of ferritin regulation and signaling, from the regulation of its intracellular levels to its effects on target cells. 

## 3. Ferritin Regulation and Signaling

As an important player in iron metabolism, ferritin levels must be tightly regulated. One of the known regulation levels occurs post transcriptionally, by the interaction of the iron responsive elements (IRE) on ferritin-encoding RNAs with iron regulatory proteins (IRP). There are two IRPs that interact with the IRE. IRP1, an iron-sulfur cluster protein, can exist in two different conformations depending on the amount of iron available. In iron-abundant environments, it adopts a cytosolic aconitase form, while in iron-deprived environments it opens to a configuration that can bind the IRE. IRP2 does not adopt different configurations, but the protein is degraded in the presence of high levels of iron. Both IRPs bind IRE under low-iron conditions and inhibit the translation of ferritin, adjusting the abundance of this protein to intracellular iron levels [21]. 

Besides iron availability, other factors can affect ferritin expression. In fact, the transcription of *Fth1* and *Ftl* is stimulated by pro-inflammatory cytokines such as interleukin (IL)-1beta [22], IL-6 [23] and tumor necrosis factor (TNF)-alpha [24] via the nuclear factor (NF)-κB pathway [25]. Interferon gamma (IFNg) and lipopolysaccharide (LPS) induce the degradation of IRP2 in a nitric oxide (NO)-dependent manner, leading to an increase in ferritin synthesis in macrophages [26,27]. IL-6 also enhances the synthesis of FTH and FTL in hepatocytes [24]. In general, the expression of FTH is more sensitive to inflammatory stimuli, whereas that of FTL responds mostly to iron levels [21,28].

The amount of intracellular ferritin is also regulated at the level of degradation, through a process called ferritinophagy. Ferritin is selectively targeted for autophagic/lysosomal degradation by the cargo nuclear receptor coactivator 4 (NCOA4), a process needed for iron release and an increase in iron availability within the cell [29,30]. TNFalpha can also contribute to the degradation of the cytosolic ferritin. Antosiewicz et al. in 2007 demonstrated that treatment with TNFalpha led to a reduction in intracellular FTL, through JNK1 signaling, with consequent increase in oxidative stress [31]. This mechanism of ferritin regulation has been associated to the induction of ferroptosis, a unique form of regulated cell death that involves increased intracellular iron availability, oxidative stress and lipid peroxidation [32,33]. Ferroptosis has been implicated in the pathological cell death associated with degenerative diseases (i.e., Alzheimer’s, Huntington’s, and Parkinson’s diseases), carcinogenesis, among others [34].

Despite being recognized mainly as an intracellular iron storage protein, ferritin is known to be present, in variable amounts, in serum. The cellular sources and the pathways of ferritin cellular export, however, are mostly unknown. Recent studies indicated that ferritin is mainly excreted from macrophages through a non-classic secretory pathway. A study by Cohen et al., using iron overloaded mice, showed the accumulation of ferritin cores in lysosomal and autophagosomal compartments of macrophages and supported the hypothesis of an active secretion of the protein [35]. An autophagy-related ferritin secretion pathway was also identified by other authors, who demonstrated the involvement of the proteins TRIM16, galectin-8 and Sec22b in this process [36]. Truman-Rosentsvit and colleagues, in turn, suggested an additional pathway for the lysosomal secretion of ferritin: the multivesicular body (MVB)–exosome pathway [37]. It was reported that both hepatocytes and macrophages have the ability to secrete ferritin, but the mechanisms by which this happens remain inconclusive [19,27]. 

The possible systemic effects of secreted ferritin are also not known. Given that there is only one protein known to mediate cellular iron export, ferroportin, it can be speculated that ferritin secreted from hepatocytes [38] and macrophages [39] could act as an additional iron-donor protein. Although serum ferritin is thought to be a poor iron carrier, given that each protein can carry more iron atoms than transferrin, it cannot be neglected as an iron delivery system. Serum ferritin has been described as consisting mostly of L subunits and very few H subunits [35]. 

In order to try to understand the possible effects of serum ferritin on target cells, there have been significant efforts to identify putative ferritin receptors. Following the observation that ferritin could modulate lymphocyte functions, it was demonstrated that mouse T cells can take up ferritin through the specific binding of the FTH peptide to T cell immunoglobulin and the mucin-domain (TIM) receptor-2 [40]. This binding leads to the endocytosis of ferritin and can mediate the uptake of iron, which will ultimately enter the cell cytosol [41]. Ferritin uptake through TIM2 was also suggested to play an important role in the central nervous system, as oligodendrocytes were shown to internalize ferritin through this pathway [42]. More recently, it was found that ferritin can also be internalized by another receptor, designated Scara5, which specifically binds L-ferritin peptides in the capsular compartment of kidney [43]. All the above-mentioned studies were done in mice. Human cells do not express TIM-2, but studies with human oligodendrocytes suggested that another receptor from the TIM family, TIM-1, was involved in ferritin uptake by these cells [44]. On the other hand, human embryonic kidney cells transfected with C-X-C chemokine receptor type 4 (CXCR4) were able to bind and internalize FTH [45]. More recently, it was shown that FTH binds to the transferrin receptor 1 (TfR1) in human cells [46]. The binding of FTH to TfR1 occurs through domains of the protein, which are distinct from those used to bind transferrin. Structural details of the interaction between the FTH and TfR1 were recently solved [47]. This binding of FTH to the TfR1 causes the uptake of FTH into endosomes and lysosomes, and it is essential for the pathways triggered by FTH [46]. Among different human hematopoietic precursors, FTH was shown to be taken up preferentially by erythroblasts, through binding to TfR1 [48]. It was shown that this internalization of FTH is important for the use of iron in the production of hemoglobulin, and therefore for the use of iron in support of erythropoiesis [49].

Overall, the physiological importance of each of these receptors is difficult to evaluate, and the molecular pathways through which ferritin can act on target cells and modulate the inflammatory or immune responses remain elusive. The possible significance of ferritin during different inflammatory conditions will be discussed in the next section. 

## 4. Ferritin in the Context of Different Diseases

Inflammation can be described as a response from the organism to a signaling damage or infection and it is characterized by heat, pain, redness, swelling, and loss of function. It can be advantageous or harmful depending on the type and duration of the stimuli [50]. It is based in an extensive diversity of physiologic and pathological processes [51]. The origin and characteristics of these stimuli are very diverse but the most important are pathogen-associated molecular patterns (PAMPs) and damage-associated molecular patterns (DAMPs), which are sensed by different molecules leading to the induction of inflammatory mediators in specific tissues [52]. Several pathways are involved in these inflammatory reactions such as the NF-κB, mitogen-activated protein kinase (MAPK) or Janus kinase/signal transducers or activators of transcription (JAK-STAT) pathways [50].

Ferritin is not only regulated by inflammatory stimuli but can also function as the enhancer of the inflammatory response. Ruddell et al. have demonstrated that FTH can activate a signaling cascade in rat’s hepatic stellate cells that leads to NF-κB activation, which will cause an increased expression of several proinflammatory mediators [25]. Interestingly, intracellular ferritin levels can also influence the production of cytokines and other mediators by immune cells. When FTH-deficient macrophages were treated with IFNg and/or bacterial LPS they had a blunted response, including a lower production of nitric oxide, IL-6 and IL1beta [18,53].

As stated before, serum ferritin is a known acute phase protein and can be used as a marker for several inflammatory pathologies, like systemic lupus erythematosus or rheumatoid arthritis [54,55]. 

In this section, we will explore the information available about the role of ferritin in different contexts of disease.

### 4.1. Cancer

Ferritin is usually detected at higher levels in the serum of cancer patients than in normal individuals. Additionally, a correlation between ferritin levels in serum and a poor clinical outcome is usually found. Different lines of research have demonstrated that ferritin has possible important roles in cancer development, through the modulation of cell proliferation [56], angiogenesis [57] and immunosuppression [58]. Tumor-associated macrophages have increased levels of ferritin, which has been suggested as having a role in tumor progression [58]. Ferritin was also found to be over-expressed in the tissues of different types of cancer [59,60,61,62,63,64]. In the case of breast cancer, the levels of ferritin were 6-fold higher than in normal breast tissue [65], which is linked with more epithelial proliferation, histopathological dedifferentiation and shorter survival [58,66]. In contrast with breast cancer, lower levels of ferritin were found in patients with colorectal cancer, as compared to healthy controls [64]. 

An interesting point that has been reported is the fact that, under hypoxic conditions, which are a hallmark of several solid tumors [59], there is an induction of ferritin expression. This effect was studied in alveolar cells from lung tumors, where ferritin expression is augmented in hypoxic conditions, independent of alterations in iron levels [63]. More recently, in vitro studies suggested that the higher levels of ferritin, particularly of the H subunit, seen in ovarian cancer, may contribute to the chemoresistance against drugs used in the treatment. This is explained by the fact that several anti-tumor drugs mediate their activity by the formation of reactive oxygen species (ROS). The higher levels of FTH can diminish the levels of ROS and then reduce the effect of the drug. The authors proposed the inhibition of FTH as a strategy to reduce the chemoresistance in ovarian cancer [62]. The role of ferritin in this pathology is still poorly understood and needs further investigation. 

Nevertheless, its role in the inflammatory processes that mediate cancer disease was recently revised. Interestingly, several lines of study found a connection between iron metabolism, tumor biology and immune surveillance. Hence, iron induces the production of ROS that can contribute to iron cell death or to malignant transformation, and then higher levels of iron are needed for cell proliferation. These processes are orchestrated by inflammatory cytokines that in turn regulate hepcidin (a key player in iron metabolism) transcription, and consequently the degradation of ferroportin and iron storage in ferritin (for a review on this topic, [60]. On the other hand, ferroptosis is an important process to facilitate cell death [61]. Tsoi et al. suggested treatment with ferroptosis inducers as an ally to actual treatments that specifically target the plasticity of melanoma-cells-associated dedifferentiation in recurrent innate and acquired resistance [67].

Importantly, in the past ten years, a new wave of research has been gaining force with the use of H-ferritin nanocarriers to deliver drugs to specific cells. This is based on the fact that FTH can specifically bind to transferrin receptor [46]. However, only FTH, and not FTL, has been shown to target cancer cells [68]. The efficacy of this new approach was already verified in murine models of gastric cancer [69] and colon cancer [70] for the delivery of the anticancer agent, doxorubicin. This gains superior interest, because it was recently shown that the FTH nanocarrier system crosses the blood brain barrier (BBB) and can kill glioma cells. This approach was based on the fact that TfR, which is the entry door of FTH nanocarrier, is highly expressed in the BBB endothelial cells and in glioma cells. Moreover, the authors found that this nanocarrier leaves the BBB by the endosome compartment, without accumulation in healthy brain tissue [68].

### 4.2. Neurodegenerative Disorders

The brain is known to have a distinct and very complex regulation of iron metabolism [71]. The failure to control iron homeostasis can lead to iron-associated neurodegenerative disorders. The role of ferritin secretion and signaling is of particular interest in neurodegenerative diseases, where it is clear that iron and ferritin misdistribution and abnormalities are an important part of the problem. As was stated before, ferritin is very important for the control of the iron levels in the cell, in order to reduce the potential production of free radicals. The failure in controlling the levels of both subunits can lead to an aberrant iron metabolism that can cause harmful events and eventually lead to several diseases. As was previously mentioned, FTH is the predominant subunit of ferritin in the brain. One example of a disease directly involving ferritin is neuroferritinopathy or hereditary ferritinopathy, an autosomal dominant disease characterized by neurodegeneration accompanied by brain iron accumulation. In neuroferritinopathy, the FTL subunit is overexpressed due to a mutation on the chromosome 19q13, leading to an abnormal ferritin structure [72,73]. With that, iron cannot be safely stored inside the cells, leading to higher levels of iron in the brain, which will induce the IRE/IRP system to produce even more ferritin. Ferritin accumulation, together with iron accumulation, will give rise to higher levels of oxidative stress that can damage brain tissue [74,75]. Amyotrophic lateral sclerosis (ALS), a neurodegenerative disease that is characterized by the degeneration of motor neurons in different tissues of the central nervous system, has also been associated with ferritin. The damage to the neurons is believed to be caused by several different factors, like oxidative damage, the disruption of key proteins or genetic factors [76]. The levels of serum ferritin are significantly higher in patients with ALS compared with healthy subjects and patients with other neurodegenerative diseases [77,78]. Although it is not clear if oxidative stress is a cause or a consequence of the disease, it is certain that ferritin may play a role. Treatments with iron chelators in combination with other molecules have already given some promising results in mice: increased levels of several regulators of mitochondrial biogenesis and metabolism could ameliorate the disease outcome [79]. Conversely, other reports indicate that ferritin levels do not correlate with survival in patients [77]. Parkinson’s disease (PD) is associated with iron overload in brain, but it is still not clear whether high iron content is the cause or an effect of PD. In a recent work, it was shown that mice lacking the expression of IRP2 have iron deposits and increased expression of FTL. Recent data showed that this is linked with aggravated neuronal apoptosis and Parkinsonism symptoms [80]. Regarding Alzheimer’s Disease (AD), recent works claim that high ferritin levels contribute to an accelerated pathology [81]. On the other hand, it was shown that the neurological impairment induced by β-amyloid is exacerbated in knockout mice for mitochondrial ferritin and that this may be related to increased levels of oxidative stress [82]. The discovery of increased iron levels in PD, AD or HF, as well as in other neurodegenerative complications, points to the relevance of the control of physiological iron concentration and compartmentalization. This is intimately linked with ferroptosis, whose characteristics of lipid peroxidation and abundant iron lead to neurodegeneration. In turn, this process can be intensified by ferritinophagy, as previously referred to, since ferritin delivery to lysosome for degradation increases iron availability and ROS production. This process can be reverted by glutathione peroxidase, if there is enough glutathione available [83,84].

In this regard, Biasiotto and colleagues proposed a model where dysfunctional ferritinophagy could be the link between autophagy impairment and iron homeostasis dysfunction in several neurodegenerative processes. Impaired autophagy due to primary causes, such as genetic mutations in genes related to the autophagic pathway, or secondary causes such as the aggregation of misfolded proteins, could also influence the selective autophagy of ferritin, inducing a mishandling of iron in the brain cells. This might contribute to a dysregulation of several cellular events in which iron is involved in the nervous system, such as myelination, neurotransmitter synthesis and mitochondrial respiration, and also oxidative stress (a hallmark of most neurodegenerative disorders), as mentioned above [85,86].

### 4.3. Cardiovascular Disease

Cardiovascular diseases (CVDs) are, according to the World Health Organization (WHO), the major cause of death globally, causing around 31% of all deaths worldwide [87]. Of the CVDs, heart failure (HF) is one of the most common and substantial problems [88]. Ferritin levels are positively correlated with other inflammation markers [89], and ferritin was suggested as a biomarker for HF in women, where the levels were significantly higher in cases with higher levels of C-reactive protein [90]. Interestingly, the levels of serum ferritin did not positively correlate with iron, as several reports have described lower serum iron levels in HF patients [91]. A role for increased intracellular iron stores has been proposed in the pathogenesis of atherosclerosis. Iron accumulation affects all cells that contribute to the atherosclerotic process, such as monocytes/macrophages, endothelial cells, vascular smooth muscle cells and platelets and it has been associated with the formation of ROS and the oxidation of lipoproteins. Ferritin has been proposed to represent a protective mechanism against atherosclerosis, both by its anti-inflammatory effect and by reducing the levels of labile iron [92]. In a very recent work, a population-based cohort study with 242,084 participants that were under clinical surveillance for more than 8 years, showed that high levels of serum ferritin do not confer an increased risk of cardiovascular disease, and questions its role as a risk factor for this disease [93].

### 4.4. Metabolic Disorders

Obesity is described by the WHO as an abnormal or excessive fat accumulation leading to health issues. The usual measure to determine if a person is obese is the body mass index (BMI). Several studies have reported that people with a high BMI have increased serum ferritin, independently of the iron stores [94,95,96]. This can be explained by the acute phase nature of serum ferritin and the role of inflammation in the development of the pathology of obesity. In this disease, there is a chronic inflammation derived from the secretion of inflammatory cytokines, like IL-6 or TNF-α by the adipose tissue. High serum ferritin levels are found in the so called “metabolically obese normal weight patients” [97]. 

Studies with several different cohorts have demonstrated that the levels of serum ferritin are directly linked with elevated fasting blood glucose, serum insulin and diagnosed diabetes, suggesting that serum ferritin may be a marker for insulin resistance [98,99,100].

The main idea is that inflammatory processes that can lead to these health issues are also responsible for the higher levels of serum ferritin.

## 5. Ferritin and Infection

The inflammatory reaction is a critical part of the host immune response to the presence of microbial pathogens. Similar to what happens in other inflammatory conditions, referred to above, ferritin is known to be increased in serum during infectious diseases, appearing in circulation as an acute phase protein, or as an inflammation and infection marker. Additionally, given the fundamental role played by ferritin in iron distribution and metabolism, it will affect host–pathogen interaction by the modulation of access to this crucial element by microbial and host cells. 

Ferritin is an evolutionarily conserved protein and it has been suggested to be involved in response to infection in different organisms, including fish and marine invertebrates. Indeed, the expression of ferritin or ferritin-homolog proteins was upregulated in different tissues in response to bacterial infections or stimulation with LPS [101,102,103,104,105,106,107,108]. Furthermore, ferritin or ferritin-homologs were shown to protect shrimp and fish from viral infections [109,110].

As a consequence of these considerations, it is conceivable that ferritin may be an important factor determining host resistance or susceptibility to infection also in mammals. In fact, increasing numbers of reports describe a role for ferritin in the outcome of various infections, including tuberculosis (TB), malaria and sepsis, among others. These reports will be discussed in this section.

### 5.1. Tuberculosis and Other Mycobacterial Infections

Despite considerable recent advances, TB continues to be a major cause of morbidity and mortality worldwide, affecting 10 million individuals and costing 1.6 million deaths annually [111]. TB, which is caused by *Mycobacterium tuberculosis* (Mtb), is transmitted among human subjects by the inhalation of aerosols containing the bacteria. As such, TB is primarily a disease of the lung, which serves as an entry point and the main site of disease manifestation. Mtb is phagocytosed by macrophages in the lung and persists and replicates mostly inside this type of cells [112]. On the other hand, approximately one fourth to one third of the human population is estimated to harbor latent Mtb infection. The niches where Mtb persists remain incompletely known and extrapulmonary, as well as pulmonary sites, have been proposed. There is an estimated 10% lifetime risk of the reactivation of latent infection, a risk that is higher in immunocompromised patients. Overall, the individual factors that determine whether a latently infected person will develop TB or not are poorly understood, and a better knowledge of innate human resistance factors would be highly valuable to improve TB management. 

The importance of the host iron status for the progress of mycobacterial infections has been extensively shown by us and by others in recent decades [113,114,115,116,117]. In general, iron overload favors the pathogen growth and the progress of the disease, while iron chelation can limit pathogen growth [118]. Accordingly, several host defense mechanisms are aimed at iron removal from circulation, reducing its availability for mycobacteria. FTH is one of the proteins that may play a role in this process. Previous work done in our group and later corroborated by others showed that primary mouse macrophages infected in vitro with *Mycobacterium avium* exhibit an increase in the expression of FTH without any relevant alteration in FTL levels. The increase in the levels of FTH is mediated by Toll-Like Receptors and is independent of TNF alpha or nitric oxide levels [119,120]. These works suggested that FTH might play a role in the outcome of mycobacterial infections in vivo. In fact, Reddy et al., using a mouse model in which *Fth1* is deleted specifically in myeloid cells, showed that FTH has a protective role in Mtb infection. Indeed, myeloid *fth1*-deficient mice had decreased survival and higher bacterial loads in their organs after aerosol infection with Mtb when compared to wild-type controls. FTH-deficiency was also associated with increased immune cell infiltration into the lungs and higher levels of pro-inflammatory cytokines such as TNFalpha, IFNg and IL-8. The authors also suggested that FTH protection against Mtb infection was associated with the maintenance of mitochondrial function [121]. We are currently investigating the role of FTH in other mycobacterial infections in vivo.

### 5.2. Malaria

Malaria, the disease caused by the protozoan parasite *Plasmodium* spp., is still a major global health problem, causing more than 430,000 deaths out of over 210 million new cases diagnosed every year [122]. Malaria parasites invade red blood cells and feed on hemoglobin. Consequently, this disease severely alters host iron metabolism and frequently leads to anemia. Variations in the host’s iron levels can have an important impact on the occurrence and the severity of the disease. Several studies have indicated that, in areas of high prevalence of iron deficiency, there was a strong protection against parasitemia- and malaria-associated mortality in childhood [123], while iron supplementation aggravated the infection [124,125]. This was confirmed in mouse models of infection, in which it was described that iron export from RBCs through ferroportin is usually increased during *Plasmodium* infection and that the conditional deletion of ferroportin in RBCs caused excessive iron accumulation, cellular impairment and increased severity of the disease [126]. Despite the impressive number of victims, more than 98% of *Plasmodium*-infected people survive, likely due to the development of disease tolerance mechanisms. A role for ferritin in this protection may be hypothesized. Epidemiological studies have found an association between malaria and increased levels of circulating ferritin. Indeed, this is an important confounding factor when trying to assess the levels of iron deficiency in malaria endemic regions [127]. However, a role for ferritin in either protection or susceptibility to malaria was not investigated in humans. Similar to TB, mouse models have been instrumental in this respect. When infected with *Plasmodium chabaudi*, mice exhibited an increase in the levels of FTH (and not FTL) in the liver. When *Fth1* expression was down-modulated, mice succumbed more rapidly to infection, in a parasite-burden-independent manner. Even more interestingly, when susceptible mice were transduced with an adenovirus coding for *Fth1* before *Plasmodium* infection, they had an increased survival compared to non-transduced mice. Again, this increase in survival was not due to a decrease in parasite load [128]. Later, the same authors demonstrated that FTH mediated host protection against malaria by preventing the development of acute renal damage during infection [129]. 

### 5.3. Sepsis

Sepsis is one of the oldest and most puzzling syndromes defying medicine. It is a set of harsh and complex physiologic, pathologic and biochemical alterations caused by infection. Although a consensus about its definition is hard to obtain, the most recent recommendation is that “Sepsis should be defined as life-threatening organ dysfunction caused by a dysregulated host response to infection” [130]. It arises when the host response to any infectious agent damages its own tissues and organs. This clinical situation is frequently underdiagnosed at initial stages, with serious consequences, as it can evolve to septic shock, which ultimately may lead to death. Sepsis still affects nearly 19 million people per year worldwide. Despite the progress made in recent decades, no specific therapy exists for this condition and 10% to 50% of the diagnosed patients die [131]. The increase in bacterial resistance to antibiotics and the lack of new effective antimicrobials clearly worsens this scenario. However, given the host-driven nature of the pathology, the need for a deeper understanding of the mechanisms involved and a detailed characterization of the evolution of the syndrome are most warranted. A possible role for ferritin in this scene is not clear but has been gaining the attention of the scientific community. 

In a series of recent publications, high levels of serum ferritin at the time of sepsis’s diagnosis were associated with an unfavorable outcome. This has been reported particularly in pediatric patients from 28 days to 18 years old [132,133,134]. Although hyperferritinemia has been increasingly acknowledged as a marker of critical illness, neither the reasons for increased ferritinemia nor the consequences of this for disease outcome are known. As mentioned before, the mechanisms of ferritin release to the circulation have been under investigation for a long time, with different studies indicating that ferritin can be released by different pathways of active exocytosis [37]. However, some authors defend that the high ferritinemia associated with critical illness results from the passive release of intracellular ferritin resulting from cell death. In line with this hypothesis, a study published last year, based on a murine model of LPS-induced sepsis, showed that ferritin release during sepsis was dependent on caspase 11 and gasdermin, which are important players in the pyroptosis type of cell death [135]. Different studies in murine models have recently added important, intriguing information regarding the potential role of ferritin in sepsis pathogenesis. 

Weis and colleagues showed, using different genetically modified mice lines with conditional deletion of the *Fth1* gene, that FTH was necessary for disease tolerance in sepsis. The protective effect of FTH was related to the decrease in oxidative stress and the preservation of gluconeogenesis, as the administration of glucose or antioxidant molecules restored, at least partially, the resistance of FTH-deficient mice. Interestingly, the authors showed that FTH expressed in parenchymal, and not in hematopoietic cells, was critical for disease tolerance [136]. This is in contrast with the results obtained with Zarjou et al. using mice deficient in FTH specifically in myeloid cells. In this case, FTH deficiency resulted in protection against death during sepsis, which the authors attributed to the compensatory increase in the expression of FTL. In fact, FTH deletion in the myeloid lineage reduced inflammation and organ injury, which resulted in lower mortality and better general outcome, while the administration of FTL to macrophages in vitro, as well as to mice in vivo, decreased the production of pro-inflammatory factors and afforded protection and resistance to the septic shock [53]. In both studies described above, the administration of apoferritin (consisting of variable amounts of H- and L-ferritin peptides, but devoid of iron) resulted in protection against sepsis lethality [53,136]. In the first, the research highlighted the immunomodulatory function of circulating ferritin by the regulation of the NF-kB pathway in vivo [53]. The latter suggests that in a sepsis model, ferritin, may act as an iron chelator [136]. Interestingly, and in line with the beneficial effects of ferritin, a protective effect of administered ferritin was shown by Lipinski P. et al., as early as 1991, in *E. coli*-induced sepsis in mice, but no discrimination was done at the time between the types of ferritin peptides that were administered [137]. Further studies are clearly warranted in order to clarify what are the sources of serum ferritin during infection are, and what, if any, is its role in the protection or exacerbation of disease. 

### 5.4. Viral Infections

Increased levels of serum ferritin have also been found in patients infected with HIV, a virus which specifically infects CD4+ T cells, weakening the immune system and making infected patients more prone to other infections and/or cancer. By the end of 2018, there were nearly 38 million people living with HIV [138].

Serum ferritin levels significantly increase very early in HIV infection, in parallel with other inflammatory mediators, including TNFa, IL-18 and type I IFN [139,140]. This early increase in serum ferritin, prior to evident alterations in systemic iron distribution, suggest that, similarly to other infectious settings, it is mainly induced by inflammation. In another curious study, Selvam et al. showed that HIV-negative infants born from HIV-positive mothers had significantly elevated ferritin levels in cord blood. This was not correlated with alterations in iron or transferrin and was instead attributed to a possible hyper-inflammatory state during pregnancy. However, the authors did not find significantly higher levels of any pro-inflammatory cytokines in the same samples [141]. The correlation between cord blood ferritin levels and any type of pathology or development problems in these infants were not investigated. 

Serum ferritin levels tend to remain high in the chronic phase of HIV infection, especially in the absence of antiretroviral therapy. Some reports show a correlation between high serum ferritin, decreased CD4 T cell numbers and mortality [139,142,143]. However, considerable variations exist between studies, and no significant differences in serum ferritin were found among HIV patients with different levels of CD4 depletion in a different cohort [144]. Another interesting observation is that in HIV patients under highly active antiretroviral therapy, high levels of serum ferritin correlate with insulin resistance (measured by the homeostatic model assessment (HOMA) method) highlighting the important relationships between inflammation, iron, glucose and lipid metabolisms, as mentioned in Section 4.4 [142].

Early in vitro studies indicated that HIV infection could alter the iron status of infected cells, namely by increasing iron accumulation [145]. However, a consensus on the detailed molecular impact of HIV infection on intracellular iron metabolism has not been reached [146]. To the best of our knowledge, the only report on the cell-intrinsic effect of HIV infection on ferritin is that of Ameglio et al. in 1993, showing that HIV-susceptible Hela-derived cell lines downregulated ferritin expression upon infection by HIV [147]. Thus, an eventual role for intracellular ferritin in HIV replication remains to be investigated. 

In contrast with HIV, patients recently infected with hepatitis B or hepatitis C virus (HBV and HCV, respectively) do not exhibit increased serum ferritin levels [139], consistent with a lower stimulation of the inflammatory response. HCV is a bloodborne virus that can cause acute or chronic hepatitis and is the major cause of liver cancer. It is estimated that more than 70 million people live with chronic HCV infection [148]. HCV infection is characterized by major alterations in iron metabolism, with increased iron deposition in the liver and paradoxically low levels of hepcidin [149]. Not surprisingly, serum ferritin levels are usually high in patients with chronic hepatitis C [149,150]. Iron accumulation in the liver contributes to pathology, supporting virus proliferation and increasing hepatic fibrosis and steatosis, and is correlated with poor outcome. Interestingly, intracellular ferritin was suggested to play a role in one of the features of HCV-induced pathology, steatosis. Liver steatosis during HCV infection occurs due to reduced production of the lipoprotein component apoB-100. In vitro studies showed that HCV-induced inhibition of apoB-100 production depends on intracellular H-ferritin and the down-regulation of Fth1 expression by SiRNA-restored apoB-100 secretion [151].

Not surprisingly, a retrospective study on patients affected by the very recent global health emergency, Covid-19, showed, once again, the presence of high levels of ferritin in the serum of patients with a more pronounced inflammatory response, associated with a poor outcome [152].

## 6. Ferritin and Other Players of Host Iron Metabolism as Possible Targets in Host-Directed Therapies 

Infectious diseases persist as an important health problem worldwide. The introduction of antibiotics clearly contributed to the reduction in disease burden, but the microbial resistance to these drugs keeps increasing and the development of new molecules lags behind. In this context, host-directed therapies appear as interesting alternatives [153,154,155]. Thus, finding host players that could be manipulated might help in the control of infection while avoiding the development of resistance associated with traditional antibiotics. The concept of host-directed therapy includes any treatment aimed at the increase in host defense strategies and/or modulation of excessive inflammation, leading to improved clinical outcome [155,156]. Host-directed therapies promote better host cellular responses to pathogens and activate both innate and adaptative responses and immunological memory. The use of host-directed therapies may include used and affordable drugs that are repurposed, instead of the developing of new compounds [155]. The current challenge is to find compounds that could interfere with host cell mechanisms and improve the immune responses or balance host responses at the site of infection. As there is increasing evidence for the role of iron in the resistance/susceptibility to infection, targeting host iron metabolism appears an obvious way to control infectious diseases. The modulation of important players of host iron metabolism has already been shown. Hepcidin mimetics and hepcidin were used to decrease the serum iron levels and, consequently, bacterial proliferation during *Vibrio vulnificus* infection in mice [157]. In another study, a calcium blocker was used to induce iron efflux from macrophages, increasing the host resistance in a murine model of *Salmonella* infection [158]. In the case of malaria, the administration of an iron chelator decreased parasite burden but did not result in improved survival when compared with the placebo group [159]. In the case of TB, iron chelators were also shown to decrease bacterial burden [115,160]. Additionally, we and others have identified iron-related proteins with protective roles during infection, namely heme-oxygenase-1 and FTH. The pharmacological modulation of the expression and/or activity of these proteins during TB or in other infection scenarios could be a way to improve infection outcome. As consequence of the increasing evidence of the role of ferritin in inflammatory processes, and particularly the need to develop new guided therapies for infectious diseases, we envisage that, in the future, ferritin might be one of those targets.

## 7. Concluding Remarks

**Figure 2:**
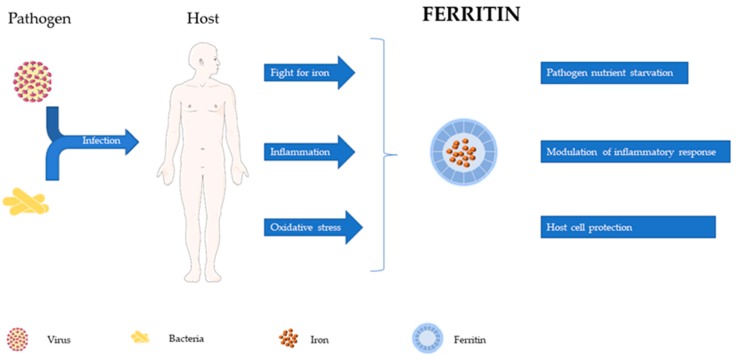
Ferritin, an inflammatory player, keeping iron at the core of pathogen–host interactions. The recognition of microbial components triggers a variety of host defense mechanisms. An important part of host defense aims at pathogen iron deprivation, achieved by the activation of iron re-distribution pathways by inflammatory mediators. The inflammatory response may, however, lead to tissue damage due to oxidative stress. Ferritin plays crucial roles in this interaction: it can decrease iron availability by intracellular sequestration, tame the inflammatory response and protect host cells from oxidative damage. Part of this scheme was elaborated with elements obtained from Servier Medical Art.

From its discovery as an intracellular iron storage protein to the recognition of its association with inflammation and infection, a long and rich evolution has occurred in our knowledge of ferritin. This review is intended to contribute to the discussion about possible roles for ferritin in the control of infection and modulation of host resistance and suggest its possible use as target in host-directed therapies (Figure 2).

## References

[1] Ganz T., Nemeth E. (2006). Regulation of iron acquisition and iron distribution in mammals. Biochim. Biophys. Acta.

[2] Gomes A.C., Mesquita G., Moreira A.C., Gomes M.S. (2018). Modulation of Iron Metabolism in Response to Infection: Twists for All Tastes. Pharmaceuticals.

[3] Arosio P., Elia L., Poli M. (2017). Ferritin, cellular iron storage and regulation. IUBMB Life.

[4] Laufberger V. (1937). Sur la cristallisation de la ferritine. Soc. Chim. Biol..

[5] Theil E.C. (1987). Ferritin: Structure, gene regulation, and cellular function in animals, plants, and microorganisms. Annu. Rev. Biochem..

[6] Recalcati S., Invernizzi P., Arosio P., Cairo G. (2008). New functions for an iron storage protein: The role of ferritin in immunity and autoimmunity. J. Autoimmun..

[7] Levi S., Corsi B., Bosisio M., Invernizzi R., Volz A., Sanford D., Arosio P., Drysdale J. (2001). A human mitochondrial ferritin encoded by an intronless gene. J. Biol. Chem..

[8] Thompson K.J., Fried M.G., Ye Z., Boyer P., Connor J.R. (2002). Regulation, mechanisms and proposed function of ferritin translocation to cell nuclei. J. Cell Sci..

[9] Arosio P., Adelman T.G., Drysdale J.W. (1978). On ferritin heterogeneity. Further evidence for heteropolymers. J. Biol. Chem..

[10] Worwood M., Brookm J.D., Cragg S.J., Hellkuhl B., Jones B.M., Perera P., Roberts S.H., Shaw D.J. (1985). Assignment of human ferritin genes to chromosomes 11 and 19q13.3→ 19qter. Hum. Genet..

[11] White K., Munro H.N. (1988). Induction of ferritin subunit synthesis by iron is regulated at both the transcriptional and translational levels. J. Biol. Chem..

[12] Lawson D.M., Artymiuk P.J., Yewdall S.J., Smith J.M., Livingstone J.C., Treffry A., Luzzago A., Levi S., Arosio P., Cesareni G. (1991). Solving the structure of human H ferritin by genetically engineering intermolecular crystal contacts. Nature.

[13] Chasteen N.D., Harrison P.M. (1999). Mineralization in ferritin: An efficient means of iron storage. J. Struct. Biol..

[14] Ferreira C., Bucchini D., Martin M.E., Levi S., Arosio P., Grandchamp B., Beaumont C. (2000). Early embryonic lethality of H ferritin gene deletion in mice. J. Biol. Chem..

[15] Arosio P., Yokota M., Drysdale J.W. (1976). Structural and immunological relationships of isoferritins in normal and malignant cells. Cancer Res..

[16] Luscieti S., Santambrogio P., Langlois d’Estaintot B., Granier T., Cozzi A., Poli M., Gallois B., Finazzi D., Cattaneo A., Levi S. (2010). Mutant ferritin L-chains that cause neurodegeneration act in a dominant-negative manner to reduce ferritin iron incorporation. J. Biol. Chem..

[17] Arosio P., Carmona F., Gozzelino R., Maccarinelli F., Poli M. (2015). The importance of eukaryotic ferritins in iron handling and cytoprotection. Biochem. J..

[18] Mesquita G., Silva T., Gomes A.C., Oliveira P.F., Alves M.G., Fernandes R., Almeida A.A., Moreira A.C., Gomes M.S. (2020). H-Ferritin is essential for macrophages’ capacity to store or detoxify exogenously added iron. Sci. Rep..

[19] Wang W., Knovich M.A., Coffman L.G., Torti F.M., Torti S.V. (2010). Serum ferritin: Past, present and future. Biochim. Biophys. Acta.

[20] Knovich M.A., Storey J.A., Coffman L.G., Torti S.V., Torti F.M. (2009). Ferritin for the clinician. Blood Rev..

[21] Torti F.M., Torti S.V. (2002). Regulation of ferritin genes and protein. Blood.

[22] Rogers J.T. (1996). Ferritin translation by interleukin-1and interleukin-6: The role of sequences upstream of the start codons of the heavy and light subunit genes. Blood.

[23] Hirayama M., Kohgo Y., Kondo H., Shintani N., Fujikawa K., Sasaki K., Kato J., Niitsu Y. (1993). Regulation of iron metabolism in HepG2 cells: A possible role for cytokines in the hepatic deposition of iron. Hepatology.

[24] Miller L.L., Miller S.C., Torti S.V., Tsuji Y., Torti F.M. (1991). Iron-independent induction of ferritin H chain by tumor necrosis factor. Proc. Natl. Acad. Sci. USA.

[25] Ruddell R.G., Hoang-Le D., Barwood J.M., Rutherford P.S., Piva T.J., Watters D.J., Santambrogio P., Arosio P., Ramm G.A. (2009). Ferritin functions as a proinflammatory cytokine via iron-independent protein kinase C zeta/nuclear factor kappaB-regulated signaling in rat hepatic stellate cells. Hepatology.

[26] Kim S., Ponka P. (2000). Effects of interferon-gamma and lipopolysaccharide on macrophage iron metabolism are mediated by nitric oxide-induced degradation of iron regulatory protein 2. J. Biol. Chem..

[27] Ghosh S., Hevi S., Chuck S.L. (2004). Regulated secretion of glycosylated human ferritin from hepatocytes. Blood.

[28] Leggett B.A., Fletcher L.M., Ramm G.A., Powell L.W., Halliday J.W. (1993). Differential regulation of ferritin H and L subunit mRNA during inflammation and long-term iron overload. J. Gastroenterol. Hepatol..

[29] Mancias J.D., Wang X., Gygi S.P., Harper J.W., Kimmelman A.C. (2014). Quantitative proteomics identifies NCOA4 as the cargo receptor mediating ferritinophagy. Nature.

[30] Hou W., Xie Y., Song X., Sun X., Lotze M.T., Zeh H.J., Kang R., Tang D. (2016). Autophagy promotes ferroptosis by degradation of ferritin. Autophagy.

[31] Antosiewicz J., Ziolkowski W., Kaczor J.J., Herman-Antosiewicz A. (2007). Tumor necrosis factor-alpha-induced reactive oxygen species formation is mediated by JNK1-dependent ferritin degradation and elevation of labile iron pool. Free Radic. Biol. Med..

[32] Dixon S.J., Lemberg K.M., Lamprecht M.R., Skouta R., Zaitsev E.M., Gleason C.E., Patel D.N., Bauer A.J., Cantley A.M., Yang W.S. (2012). Ferroptosis: An iron-dependent form of nonapoptotic cell death. Cell.

[33] Gao M., Monian P., Pan Q., Zhang W., Xiang J., Jiang X. (2016). Ferroptosis is an autophagic cell death process. Cell Res..

[34] Stockwell B.R., Friedmann Angeli J.P., Bayir H., Bush A.I., Conrad M., Dixon S.J., Fulda S., Gascón S., Hatzios S.K., Kagan V.E. (2017). Ferroptosis: A Regulated Cell Death Nexus Linking Metabolism, Redox Biology, and Disease. Cell.

[35] Cohen L.A., Gutierrez L., Weiss A., Leichtmann-Bardoogo Y., Zhang D.L., Crooks D.R., Sougrat R., Morgenstern A., Galy B., Hentze M.W. (2010). Serum ferritin is derived primarily from macrophages through a nonclassical secretory pathway. Blood.

[36] Kimura T., Jia J., Kumar S., Choi S.W., Gu Y., Mudd M., Dupont N., Jiang S., Peters R., Farzam F. (2017). Dedicated SNAREs and specialized TRIM cargo receptors mediate secretory autophagy. EMBO J..

[37] Truman-Rosentsvit M., Berenbaum D., Spektor L., Cohen L.A., Belizowsky-Moshe S., Lifshitz L., Ma M., Li W., Kesselman E., Abutbul-Ionita I. (2018). Ferritin is secreted via 2 distinct nonclassical vesicular pathways. Blood.

[38] Sibille J.C., Kondo H., Aisen P. (1988). Interactions between isolated hepatocytes and Kupffer cells in iron metabolism: A possible role for ferritin as an iron carrier protein. Hepatology.

[39] Leimberg J.M., Konijn A.M., Fibach E. (2005). Macrophages promote development of human erythroid precursors in transferrin-free culture medium. Hematology.

[40] Chen T.T., Li L., Chung D.H., Allen C.D., Torti S.V., Torti F.M., Cyster J.G., Chen C.Y., Brodsky F.M., Niemi E.C. (2005). TIM-2 is expressed on B cells and in liver and kidney and is a receptor for H-ferritin endocytosis. J. Exp. Med..

[41] Han J., Seaman W.E., Di X., Wang W., Willingham M., Torti F.M., Torti S.V. (2011). Iron uptake mediated by binding of H-ferritin to the TIM-2 receptor in mouse cells. PLoS ONE.

[42] Todorich B., Zhang X., Slagle-Webb B., Seaman W.E., Connor J.R. (2008). Tim-2 is the receptor for H-ferritin on oligodendrocytes. J. Neurochem..

[43] Li J.Y., Paragas N., Ned R.M., Qiu A., Viltard M., Leete T., Drexler I.R., Chen X., Sanna-Cherchi S., Mohammed F. (2009). Scara5 is a ferritin receptor mediating non-transferrin iron delivery. Dev. Cell.

[44] Chiou B., Lucassen E., Sather M., Kallianpur A., Connor J. (2018). Semaphorin4A and H-ferritin utilize Tim-1 on human oligodendrocytes: A novel neuro-immune axis. Glia.

[45] Li R., Luo C., Mines M., Zhang J., Fan G.H. (2006). Chemokine CXCL12 induces binding of ferritin heavy chain to the chemokine receptor CXCR4, alters CXCR4 signaling, and induces phosphorylation and nuclear translocation of ferritin heavy chain. J. Biol. Chem..

[46] Li L., Fang C.J., Ryan J.C., Niemi E.C., Lebrón J.A., Björkman P.J., Arase H., Torti F.M., Torti S.V., Nakamura M.C. (2010). Binding and uptake of H-ferritin are mediated by human transferrin receptor-1. Proc. Natl. Acad. Sci. USA.

[47] Montemiglio L.C., Testi C., Ceci P., Falvo E., Pitea M., Savino C., Arcovito A., Peruzzi G., Baiocco P., Mancia F. (2019). Cryo-EM structure of the human ferritin-transferrin receptor 1 complex. Nat. Commun..

[48] Sakamoto S., Kawabata H., Masuda T., Uchiyama T., Mizumoto C., Ohmori K., Koeffler H.P., Kadowaki N., Takaori-Kondo A. (2015). H-Ferritin Is Preferentially Incorporated by Human Erythroid Cells through Transferrin Receptor 1 in a Threshold-Dependent Manner. PLoS ONE.

[49] Leimberg M.J., Prus E., Konijn A.M., Fibach E. (2008). Macrophages function as a ferritin iron source for cultured human erythroid precursors. J. Cell. Biochem..

[50] Chen L., Deng H., Cui H., Fang J., Zuo Z., Deng J., Li Y., Wang X., Zhao L. (2018). Inflammatory responses and inflammation-associated diseases in organs. Oncotarget.

[51] Medzhitov R. (2008). Origin and physiological roles of inflammation. Nature.

[52] Van Linthout S., Tschope C. (2017). Inflammation—Cause or Consequence of Heart Failure or Both?. Curr. Heart Fail. Rep..

[53] Zarjou A., Black L.M., McCullough K.R., Hull T.D., Esman S.K., Boddu R., Varambally S., Chandrashekar D.S., Feng W., Arosio P. (2019). Ferritin Light Chain Confers Protection Against Sepsis-Induced Inflammation and Organ Injury. Front. Immunol..

[54] Vanarsa K., Ye Y., Han J., Xie C., Mohan C., Wu T. (2012). Inflammation associated anemia and ferritin as disease markers in SLE. Arthritis Res. Ther..

[55] Seyhan S., Pamuk Ö.N., Pamuk G.E., Çakır N. (2014). The correlation between ferritin level and acute phase parameters in rheumatoid arthritis and systemic lupus erythematosus. Eur. J. Rheumatol..

[56] Li S. (2010). Identification of iron-loaded ferritin as an essential mitogen for cell proliferation and postembryonic development in Drosophila. Cell Res..

[57] Tesfay L., Huhn A.J., Hatcher H., Torti F.M., Torti S.V. (2012). Ferritin blocks inhibitory effects of two-chain high molecular weight kininogen (HKa) on adhesion and survival signaling in endothelial cells. PLoS ONE.

[58] Alkhateeb A.A., Connor J.R. (2013). The significance of ferritin in cancer: Anti-oxidation, inflammation and tumorigenesis. Biochim. Biophys. Acta.

[59] Parks S.K., Cormerais Y., Pouyssegur J. (2017). Hypoxia and cellular metabolism in tumour pathophysiology. J. Physiol..

[60] Pfeifhofer-Obermair C., Tymoszuk P., Petzer V., Weiss G., Nairz M. (2018). Iron in the Tumor Microenvironment-Connecting the Dots. Front. Oncol..

[61] Proneth B., Conrad M. (2019). Ferroptosis and necroinflammation, a yet poorly explored link. Cell Death Differ..

[62] Salatino A., Aversa I., Battaglia A.M., Sacco A., Di Vito A., Santamaria G., Chirillo R., Veltri P., Tradigo G., Di Cello A. (2019). H-Ferritin Affects Cisplatin-Induced Cytotoxicity in Ovarian Cancer Cells through the Modulation of ROS. Oxid. Med. Cell. Longev..

[63] Smith J.J., O’Brien-Ladner A.R., Kaiser C.R., Wesselius L.J. (2003). Effects of hypoxia and nitric oxide on ferritin content of alveolar cells. J. Lab. Clin. Med..

[64] Feng Z., Chen J.W., Feng J.H., Shen F., Cai W.S., Cao J., Xu B. (2015). The association between serum ferritin with colorectal cancer. Int. J. Clin. Exp. Med..

[65] Weinstein R.E., Bond B.H., Silberberg B.K. (1982). Tissue ferritin concentration in carcinoma of the breast. Cancer.

[66] Shpyleva S.I., Tryndyak V.P., Kovalchuk O., Starlard-Davenport A., Chekhun V.F., Beland F.A., Pogribny I.P. (2011). Role of ferritin alterations in human breast cancer cells. Breast Cancer Res. Treat..

[67] Tsoi J., Robert L., Paraiso K., Galvan C., Sheu K.M., Lay J., Wong D.J.L., Atefi M., Shirazi R., Wang X. (2018). Multi-stage Differentiation Defines Melanoma Subtypes with Differential Vulnerability to Drug-Induced Iron-Dependent Oxidative Stress. Cancer Cell.

[68] Fan K., Jia X., Zhou M., Wang K., Conde J., He J., Tian J., Yan X. (2018). Ferritin Nanocarrier Traverses the Blood Brain Barrier and Kills Glioma. ACS Nano.

[69] Cheng X., Fan K., Wang L., Ying X., Sanders A.J., Guo T., Xing X., Zhou M., Du H., Hu Y. (2020). TfR1 binding with H-ferritin nanocarrier achieves prognostic diagnosis and enhances the therapeutic efficacy in clinical gastric cancer. Cell Death Dis..

[70] Liang M., Fan K., Zhou M., Duan D., Zheng J., Yang D., Feng J., Yan X. (2014). H-ferritin-nanocaged doxorubicin nanoparticles specifically target and kill tumors with a single-dose injection. Proc. Natl. Acad. Sci. USA.

[71] Madsen E., Gitlin J.D. (2007). Copper and iron disorders of the brain. Annu. Rev. Neurosci..

[72] Levi S., Cozzi A., Arosio P. (2005). Neuroferritinopathy: A neurodegenerative disorder associated with L-ferritin mutation. Best Pract. Res. Clin. Haematol..

[73] Curtis A.R., Fey C., Morris C.M., Bindoff L.A., Ince P.G., Chinnery P.F., Coulthard A., Jackson M.J., Jackson A.P., McHale D.P. (2001). Mutation in the gene encoding ferritin light polypeptide causes dominant adult-onset basal ganglia disease. Nat. Genet..

[74] Vidal R., Miravalle L., Gao X., Barbeito A.G., Baraibar M.A., Hekmatyar S.K., Widel M., Bansal N., Delisle M.B., Ghetti B. (2008). Expression of a mutant form of the ferritin light chain gene induces neurodegeneration and iron overload in transgenic mice. J. Neurosci..

[75] Barbeito A.G., Garringer H.J., Baraibar M.A., Gao X., Arredondo M., Núñez M.T., Smith M.A., Ghetti B., Vidal R. (2009). Abnormal iron metabolism and oxidative stress in mice expressing a mutant form of the ferritin light polypeptide gene. J. Neurochem..

[76] Redler R.L., Dokholyan N.V. (2012). The complex molecular biology of amyotrophic lateral sclerosis (ALS). Prog. Mol. Biol. Transl. Sci..

[77] Su X.W., Clardy S.L., Stephens H.E., Simmons Z., Connor J.R. (2015). Serum ferritin is elevated in amyotrophic lateral sclerosis patients. Amyotroph. Lateral Scler. Front. Degener..

[78] Yu J., Wang N., Qi F., Wang X., Zhu Q., Lu Y., Zhang H., Che F., Li W. (2018). Serum ferritin is a candidate biomarker of disease aggravation in amyotrophic lateral sclerosis. Biomed. Rep..

[79] Golko-Perez S., Mandel S., Amit T., Kupershmidt L., Youdim M.B., Weinreb O. (2016). Additive Neuroprotective Effects of the Multifunctional Iron Chelator M30 with Enriched Diet in a Mouse Model of Amyotrophic Lateral Sclerosis. Neurotox. Res..

[80] Ci Y.Z., Li H., You L.H., Jin Y., Zhou R., Gao G., Hoi M.P.M., Wang C., Chang Y.Z., Yu P. (2020). Iron overload induced by IRP2 gene knockout aggravates symptoms of Parkinson’s disease. Neurochem. Int..

[81] Bester J., Buys A.V., Lipinski B., Kell D.B., Pretorius E. (2013). High ferritin levels have major effects on the morphology of erythrocytes in Alzheimer’s disease. Front. Aging Neurosci..

[82] Wang P., Wu Q., Wu W., Li H., Guo Y., Yu P., Gao G., Shi Z., Zhao B., Chang Y.Z. (2017). Mitochondrial Ferritin Deletion Exacerbates beta-Amyloid-Induced Neurotoxicity in Mice. Oxid. Med. Cell. Longev..

[83] Tang M., Chen Z., Wu D., Chen L. (2018). Ferritinophagy/ferroptosis: Iron-related newcomers in human diseases. J. Cell. Physiol..

[84] Muhoberac B.B., Vidal R. (2019). Iron, Ferritin, Hereditary Ferritinopathy, and Neurodegeneration. Front. Neurosci..

[85] Biasiotto G., Di Lorenzo D., Archetti S., Zanella I. (2016). Iron and Neurodegeneration: Is Ferritinophagy the Link?. Mol. Neurobiol..

[86] Quiles Del Rey M., Mancias J.D. (2019). NCOA4-Mediated Ferritinophagy: A Potential Link to Neurodegeneration. Front. Neurosci..

[87] WHO (2017). Cardiovascular Diseases (CVDs).

[88] Timmis A., Townsend N., Gale C.P., Torbica A., Lettino M., Petersen S.E., Mossialos E.A., Maggioni A.P., Kazakiewicz D., May H.T. (2020). European Society of Cardiology: Cardiovascular Disease Statistics 2019. Eur. Heart J..

[89] Perticone M., Zito R., Miceli S., Pinto A., Suraci E., Greco M., Gigliotti S., Hribal M.L., Corrao S., Sesti G. (2019). Immunity, Inflammation and Heart Failure: Their Role on Cardiac Function and Iron Status. Front. Immunol..

[90] Williams M.J., Poulton R., Williams S. (2002). Relationship of serum ferritin with cardiovascular risk factors and inflammation in young men and women. Atherosclerosis.

[91] Von Haehling S., Ebner N., Evertz R., Ponikowski P., Anker S.D. (2019). Iron Deficiency in Heart Failure: An Overview. JACC Heart Fail..

[92] Kraml P. (2017). The role of iron in the pathogenesis of atherosclerosis. Physiol. Res..

[93] Reyes C., Pons N.A., Reñones C.R., Gallisà J.B., Val V.A., Tebé C., Mateo G.F. (2020). Association between serum ferritin and acute coronary heart disease: A population-based cohort study. Atherosclerosis.

[94] Alam F., Memon A.S., Fatima S.S. (2015). Increased Body Mass Index may lead to Hyperferritinemia Irrespective of Body Iron Stores. Pak. J. Med. Sci..

[95] Bonora M., Wieckowsk M.R., Chinopoulos C., Kepp O., Kroemer G., Galluzzi L., Pinton P. (2015). Molecular mechanisms of cell death: Central implication of ATP synthase in mitochondrial permeability transition. Oncogene.

[96] Khan A., Khan W.M., Ayub M., Humayun M., Haroon M. (2016). Ferritin Is a Marker of Inflammation rather than Iron Deficiency in Overweight and Obese People. J. Obes..

[97] Kim J.W., Kim D.H., Roh Y.K., Ju S.Y., Nam H.Y., Nam G.E., Kim D.W., Lee S.H., Lee C.W., Han K. (2015). Serum Ferritin Levels Are Positively Associated With Metabolically Obese Normal Weight: A Nationwide Population-Based Study. Medicine.

[98] Ashourpour M., Djalali M., Djazayery A., Eshraghian M.R., Taghdir M., Saedisomeolia A. (2010). Relationship between serum ferritin and inflammatory biomarkers with insulin resistance in a Persian population with type 2 diabetes and healthy people. Int. J. Food Sci. Nutr..

[99] Ford E.S., Cogswell M.E. (1999). Diabetes and serum ferritin concentration among U.S. adults. Diabetes Care.

[100] Tuomainen T.P., Nyyssönen K., Salonen R., Tervahauta A., Korpela H., Lakka T., Kaplan G.A., Salonen J.T. (1997). Body iron stores are associated with serum insulin and blood glucose concentrations. Population study in 1013 eastern Finnish men. Diabetes Care.

[101] Martinez D., Oyarzún R., Vargas-Lagos C., Pontigo J.P., Soto-Dávila M., Saravia J., Romero A., Núñez J.J., Yáñez A.J., Vargas-Chacoff L. (2017). Identification, characterization and modulation of ferritin-H in the sub-Antarctic Notothenioid Eleginops maclovinus challenged with Piscirickettsia salmonis. Dev. Comp. Immunol..

[102] Neves J.V., Wilson J.M., Rodrigues P.N. (2009). Transferrin and ferritin response to bacterial infection: The role of the liver and brain in fish. Dev. Comp. Immunol..

[103] Sun S., Zhu J., Ge X., Zhang W. (2016). Molecular characterization and gene expression of ferritin in blunt snout bream (Megalobrama amblycephala). Fish. Shellfish Immunol..

[104] Chen G., Zhang C., Wang Y., Guo C., Sang F., Wang C. (2016). Identification and characterization of a ferritin gene involved in the immune defense response of scallop Chlamys farreri. Fish. Shellfish Immunol..

[105] He S., Peng K., Hong Y., Wang J., Sheng J., Gu Q. (2013). Molecular properties and immune defense of two ferritin subunits from freshwater pearl mussel, Hyriopsis schlegelii. Fish. Shellfish Immunol..

[106] Liu Q.N., Xin Z.Z., Liu Y., Wang Z.F., Chen Y.J., Zhang D.Z., Jiang S.H., Chai X.Y., Zhou C.L., Tang B.P. (2017). A ferritin gene from Procambarus clarkii, molecular characterization and in response to heavy m.etal stress and lipopolysaccharide challenge. Fish. Shellfish Immunol..

[107] Ren C., Chen T., Jiang X., Wang Y., Hu C. (2014). Identification and functional characterization of a novel ferritin subunit from the tropical sea cucumber, Stichopus monotuberculatus. Fish. Shellfish Immunol..

[108] Chen S., Wua C., Xiea Y., Wua Y., Daia S., Wanga X., Lib R., Ye J. (2020). Molecular cloning, characterization and expression modulation of four ferritins in black carp Mylopharyngodon piceus in response to Aeromonas hydrophila challenge. Aquac. Rep..

[109] Chen X.X., Li Y.Y., Chang X.J., Xie X.L., Liang Y.T., Wang K.J., Zheng W.Y., Liu H.P. (2018). A CqFerritin protein inhibits white spot syndrome virus infection via regulating iron ions in red claw crayfish Cherax quadricarinatus. Dev. Comp. Immunol..

[110] Ye T., Wu X., Wu W., Dai C., Yuan J. (2015). Ferritin protect shrimp Litopenaeus vannamei from WSSV infection by inhibiting virus replication. Fish. Shellfish Immunol..

[111] WHO (2018). Global Tuberculosis Report 2018.

[112] Podinovskaia M., Lee W., Caldwell S., Russell D.G. (2013). Infection of macrophages with Mycobacterium tuberculosis induces global modifications to phagosomal function. Cell. Microbiol..

[113] Gomes M.S., Dom G., Pedrosa J., Boelaert J.R., Appelberg R. (1999). Effects of iron deprivation on Mycobacterium avium growth. Tuber. Lung Dis..

[114] Gomes M.S., Appelberg R. (1998). Evidence for a link between iron metabolism and Nramp1 gene function in innate resistance against Mycobacterium avium. Immunology.

[115] Louis N., Maslo C., Boelaert J., Bonnafous P., Trnffot-Pernot C., Baohong J., Grosset J. (1999). Impact of iron loading and iron chelation on murine tuberculosis. Clin. Microbiol. Infect..

[116] Gomes-Pereira S., Rodrigues P.N., Appelberg R., Gomes M.S. (2008). Increased susceptibility to Mycobacterium avium in hemochromatosis protein HFE-deficient mice. Infect. Immun..

[117] Gomes M.S., Boelaert J.R., Appelberg R. (2001). Role of iron in experimental Mycobacterium avium infection. J. Clin. Virol..

[118] Moniz T., Silva D., Silva T., Gomes M.S., Rangel M. (2015). Antimycobacterial activity of rhodamine 3,4-HPO iron chelators against Mycobacterium avium: Analysis of the contribution of functional groups and of chelator’s combination with ethambutol. MedChemComm.

[119] Silva-Gomes S., Bouton C., Silva T., Santambrogio P., Rodrigues P., Appelberg R., Gomes M.S. (2013). Mycobacterium avium infection induces H-ferritin expression in mouse primary macrophages by activating Toll-like receptor 2. PLoS ONE.

[120] Abreu R., Quinn F., Giri P.K. (2018). Role of the hepcidin-ferroportin axis in pathogen-mediated intracellular iron sequestration in human phagocytic cells. Blood Adv..

[121] Reddy V.P., Chinta K.C., Saini V., Glasgow J.N., Hull T.D., Traylor A., Rey-Stolle F., Soares M.P., Madansein R., Rahman M.A. (2018). Ferritin H Deficiency in Myeloid Compartments Dysregulates Host Energy Metabolism and Increases Susceptibility to Mycobacterium tuberculosis Infection. Front. Immunol..

[122] WHO (2019). World Malaria Report 2019.

[123] Gwamaka M., Kurtis J.D., Sorensen B.E., Holte S., Morrison R., Mutabingwa T.K., Fried M., Duffy P.E. (2012). Iron deficiency protects against severe Plasmodium falciparum malaria and death in young children. Clin. Infect. Dis..

[124] Clark M.A., Goheen M.M., Fulford A., Prentice A.M., Elnagheeb M.A., Patel J., Fisher N., Taylor S.M., Kasthuri R., Cerami C. (2014). Host iron status and iron supplementation mediate susceptibility to erythrocytic stage Plasmodium falciparum. Nat. Commun..

[125] Clark M.A., Goheen M.M., Cerami C. (2014). Influence of host iron status on Plasmodium falciparum infection. Front. Pharmacol..

[126] Zhang D.L., Wu J., Shah B.N., Greutélaers K.C., Ghosh M.C., Ollivierre H., Su X.Z., Thuma P.E., Bedu-Addo G., Mockenhaupt F.P. (2018). Erythrocytic ferroportin reduces intracellular iron accumulation, hemolysis, and malaria risk. Science.

[127] Barffour M.A., Schulze K.J., Coles C.L., Chileshe J., Kalungwana N., Siamusantu W., Arguello M., Moss W.J., West K.P., Palmer A.C. (2018). Malaria exacerbates inflammation-associated elevation in ferritin and soluble transferrin receptor with only modest effects on iron deficiency and iron deficiency anaemia among rural Zambian children. Trop. Med. Int. Health.

[128] Gozzelino R., Andrade B.B., Larsen R., Luz N.F., Vanoaica L., Seixas E., Coutinho A., Cardoso S., Rebelo S., Poli M. (2012). Metabolic adaptation to tissue iron overload confers tolerance to malaria. Cell Host Microbe.

[129] Ramos S., Ramos S., Carlos A.R., Sundaram B., Jeney V., Ribeiro A., Gozzelino R., Bank C., Gjini E., Braza F. (2019). Renal control of disease tolerance to malaria. Proc. Natl. Acad. Sci. USA.

[130] Singer M., Deutschman C.S., Seymour C.W., Shankar-Hari M., Annane D., Bauer M., Bellomo R., Bernard G.R., Chiche J.D., Coopersmith C.M. (2016). The Third International Consensus Definitions for Sepsis and Septic Shock (Sepsis-3). JAMA.

[131] Angus D.C., van der Poll T. (2013). Severe sepsis and septic shock. N. Engl. J. Med..

[132] Tonial C.T., Garcia P.C.R., Schweitzer L.C., Costa C.A.D., Bruno F., Fiori H.H., Einloft P.R., Garcia R.B., Piva J.P. (2017). Cardiac dysfunction and ferritin as early markers of severity in pediatric sepsis. J. Pediatr..

[133] Garcia P.C., Longhi F., Branco R.G., Piva J.P., Lacks D., Tasker R.C. (2007). Ferritin levels in children with severe sepsis and septic shock. Acta Paediatr..

[134] Carcillo J.A., Halstead E.S., Hall M.W., Nguyen T.C., Reeder R., Aneja R., Shakoory B., Simon D. (2017). Three Hypothetical Inflammation Pathobiology Phenotypes and Pediatric Sepsis-Induced Multiple Organ Failure Outcome. Pediatr. Crit. Care Med..

[135] Wang D., Yu S., Zhang Y., Huang L., Luo R., Tang Y., Zhao K., Lu B. (2019). Caspse-11-GSDMD pathway is required for serum ferritin secretion in sepsis. Clin. Immunol..

[136] Weis S., Carlos A.R., Moita M.R., Singh S., Blankenhaus B., Cardoso S., Larsen R., Rebelo S., Schäuble S., Del Barrio L. (2017). Metabolic Adaptation Establishes Disease Tolerance to Sepsis. Cell.

[137] Lipinski P., Jarzabek Z., Broniek S., Zagulski T. (1991). Protective effect of tissue ferritins in experimental Escherichia coli infection of mice in vivo. Int. J. Exp. Pathol..

[138] WHO (2018). Progress Report on HIV, Viral Hepatitis and Sexually Transmitted Infections 2019.

[139] Armitage A.E., Stacey A.R., Giannoulatou E., Marshall E., Sturges P., Chatha K., Smith N.M., Huang X., Xu X., Pasricha S.R. (2014). Distinct patterns of hepcidin and iron regulation during HIV-1, HBV, and HCV infections. Proc. Natl. Acad. Sci. USA.

[140] Babiker Z.O., Wingfield T., Galloway J., Snowden N., Ustianowski A. (2015). Extreme elevation of ferritin and creatine kinase in primary infection with HIV-1. Int. J. STD AIDS.

[141] Selvam A., Buhimschi I.A., Makin J.D., Pattinson R.C., Anderson R., Forsyth B.W. (2015). Hyperferritinemia and markers of inflammation and oxidative stress in the cord blood of HIV-exposed, uninfected (HEU) infants. HIV Med..

[142] Wisaksana R., Sumantri R., Indrati A.R., Zwitser A., Jusuf H., de Mast Q., van Crevel R., van der Ven A. (2011). Anemia and iron homeostasis in a cohort of HIV-infected patients in Indonesia. BMC Infect. Dis..

[143] Lopez-Calderon C., Palacios R., Cobo A., Nuño E., Ruiz J., Márquez M., Santos J. (2015). Serum ferritin in HIV-positive patients is related to immune deficiency and inflammatory activity. Int. J. STD AIDS.

[144] Obirikorang C., Issahaku R.G., Osakunor D.N., Osei-Yeboah J. (2016). Anaemia and Iron Homeostasis in a Cohort of HIV-Infected Patients: A Cross-Sectional Study in Ghana. AIDS Res. Treat..

[145] Drakesmith H., Chen N., Ledermann H., Screaton G., Townsend A., Xu X.N. (2005). HIV-1 Nef down-regulates the hemochromatosis protein HFE, manipulating cellular iron homeostasis. Proc. Natl. Acad. Sci. USA.

[146] Matheson N.J., Greenwood E.J., Lehner P.J. (2016). Manipulation of immunometabolism by HIV-accessories to the crime?. Curr. Opin. Virol..

[147] Ameglio F., Tilocca F., Arca M.V., Alemanno L., Dolei A. (1993). Ferritin downregulation in HIV-infected cells. AIDS Res. Hum. Retrovir..

[148] WHO (2017). Global Hepatitis Report 2017.

[149] Hino K., Nishina S., Sasaki K., Hara Y. (2019). Mitochondrial damage and iron metabolic dysregulation in hepatitis C virus infection. Free Radic. Biol. Med..

[150] Batsaikhan B., Gantumur G., Huang C.I., Yeh M.L., Huang C.F., Lin Z.Y., Chen S.C., Huang J.F., Yu M.L., Chuang W.L. (2019). Elevated serum ferritin level associated with hepatic steatosis and fibrosis in hepatitis C virus-infected patients. J. Chin. Med. Assoc..

[151] Mancone C., Montaldo C., Santangelo L., Di Giacomo C., Costa V., Amicone L., Ippolito G., Pucillo L.P., Alonzi T., Tripodi M. (2012). Ferritin heavy chain is the host factor responsible for HCV-induced inhibition of apoB-100 production and is required for efficient viral infection. J. Proteome Res..

[152] Zhou F., Yu T., Du R., Fan G., Liu Y., Liu Z., Xiang J., Wang Y., Song B., Gu X. (2020). Clinical course and risk factors for mortality of adult inpatients with COVID-19 in Wuhan, China: A retrospective cohort study. Lancet.

[153] Bento C.M., Gomes M.S., Silva T. (2020). Looking beyond Typical Treatments for Atypical Mycobacteria. Antibiotics.

[154] Kaufmann S.H.E., Dorhoi A., Hotchkiss R., Bartenschlager R. (2018). Host-directed therapies for bacterial and viral infections. Nat. Rev. Drug Discov..

[155] Zumla A., Rao M., Wallis R.S., Kaufmann S.H., Rustomjee R., Mwaba P., Vilaplana C., Yeboah-Manu D., Chakaya J., Ippolito G. (2016). Host-directed therapies for infectious diseases: Current status, recent progress, and future prospects. Lancet Infect. Dis..

[156] Chiang C.Y., Chiang C.Y., Uzoma I., Moore R.T., Gilbert M., Duplantier A.J., Panchal R.G. (2018). Mitigating the Impact of Antibacterial Drug Resistance through Host-Directed Therapies: Current Progress, Outlook, and Challenges. MBio.

[157] Arezes J., Jung G., Gabayan V., Valore E., Ruchala P., Gulig P.A., Ganz T., Nemeth E., Bulut Y. (2015). Hepcidin-induced hypoferremia is a critical host defense mechanism against the siderophilic bacterium Vibrio vulnificus. Cell Host Microbe.

[158] Mair S.M., Nairz M., Bellmann-Weiler R., Muehlbacher T., Schroll A., Theurl I., Moser P.L., Talasz H., Fang F.C., Weiss G. (2011). Nifedipine affects the course of Salmonella enterica serovar Typhimurium infection by modulating macrophage iron homeostasis. J. Infect. Dis..

[159] Gordeuk V., Thuma P., Brittenham G., McLaren C., Parry D., Backenstose A., Biemba G., Msiska R., Holmes L., McKinley E. (1992). Effect of iron chelation therapy on recovery from deep coma in children with cerebral malaria. N. Engl. J. Med..

[160] Schaible U.E., Collins H.L., Priem F., Kaufmann S.H. (2002). Correction of the iron overload defect in beta-2-microglobulin knockout mice by lactoferrin abolishes their increased susceptibility to tuberculosis. J. Exp. Med..

